# Clinical and radiographic performance of preformed zirconia crowns and stainless-steel crowns in permanent first molars: 18-month results of a prospective, randomized trial

**DOI:** 10.1186/s12903-023-03501-1

**Published:** 2023-11-03

**Authors:** Nazli Geduk, Merve Ozdemir, Gizem Erbas Unverdi, Elif Ballikaya, Zafer C. Cehreli

**Affiliations:** 1 Denturla Oral and Dental Health Polyclinic, Urla, 35430 Izmir Turkey; 2 Department of Pediatric Dentistry, Faculty of Dentistry, Lokman Hekim University, Sogutozu, Ankara, 06510 Turkey; 3 Department of Pediatric Dentistry, Faculty of Dentistry, Hacettepe University, Sihhiye, Ankara, 06100 Turkey

**Keywords:** Children, Permanent molars, Pediatric crowns, Preformed Zirconia crowns, Stainless Steel crowns, Randomized Controlled Trial

## Abstract

**Background:**

The treatment of young permanent first molars with extensive carious tissue loss may often require restoration with preformed crowns. This study compared the clinical and radiographic performance of stainless-steel crowns (SSCs) and preformed zirconia crowns (ZCs).

**Methods:**

Forty-eight molar incisor hypomineralisation (MIH)- or caries-affected permanent molars in 20 healthy patients between 6–13-year-old were randomly divided into ZC and SSC groups (n = 24 teeth/group) in a split-mouth design. The oral hygiene levels of patients were assessed using Greene and Vermillion simplified oral hygiene index (OHI-S). Plaque accumulation and gingival health were evaluated using the Silness&Löe plaque index (PI) and Löe&Silness gingival index (GI), respectively. Clinical retention, marginal extension level, marginal adaptation of crowns and wear of the antagonist teeth were assessed at baseline, 1, 6, 12 and 18 months. The radiological assessments for evaluating the marginal adaptation of crowns and periapical pathology of crowned teeth were performed at 6 and 12 months. The data were analyzed using Kaplan–Meier analysis, Mann–Whitney U test, and two-way ANOVA.

**Results:**

A total of forty teeth in 17 children were evaluated for 18 months. ZCs had significantly lower gingival and plaque index values than teeth restored with SSCs during all evaluation periods (p < 0.05). Neither crown type resulted in clinically-detectable wear on opposing dentition or periapical pathology. One ZC was lost at 13 months, while all SSCs survived in function clinically. The cumulative survival rates of ZCs and SSCs were 95.2% and 100% respectively.

**Conclusions:**

Both ZCs and SSCs showed high clinical retention rates in young permanent molars. ZCs had lower plaque accumulation and better gingival health than SSCs, which were consistently associated with mild gingival inflammation.

**Clinical Trial Registration Number:**

NCT05049694.

## Introduction

The first permanent molars (FPMs) are the most caries-prone teeth [1]. They erupt within the oral cavity relatively early and are highly susceptible to both plaque accumulation and caries progression until the teeth achieve full occlusion, which can take up to a year. [1]. During this period, parents may believe that these are primary molars and that they will fall out soon [2]. In addition, the first permanent molars are susceptible to various developmental defects including molar-incisor hypomineralisation (MIH) and hypoplasia [3]. In young individuals who are at high risk for caries and have poor oral hygiene, these variables may contribute to extensive and/or multisurface carious lesions at an early age. The presence and extent of enamel defects, as well as post-eruptive breakdown, also affect the prognosis of these teeth [4]. However, large carious lesions can also develop in teeth with minimal signs of a pre-existing enamel defect [4]. The National Health Services Dental Clinical Guidance defines FPMs that have moderate to severe molar incisor hypomineralisation (MIH), advanced or unrestorable caries, pulpitis with reversible or irreversible symptoms, radiographically evident pulpal involvement or periradicular pathology as *FPMs with poor prognosis* [5]. The prevalence of FPMs with poor prognosis was reported to be 35.16% in a recent study [6], and stainless steel crowns or zirconia crowns were recommended following the treatments of pulp capping and pulpotomy/pulpectomy.

At present, there is a lack of consensus regarding the optimal approach for managing compromised first permanent molars (FPMs) in children. The existing evidence is inconclusive, and there is no universally accepted treatment that is considered the superior choice [7]. The treatment options are often determined by a number of criteria including the patient’s age, compliance, oral hygiene, existing malocclusion, future orthodontic needs, parental attitude, and the tooth’s restorability [7–9]. The use of stainless-steel permanent molar crowns (SSCs) has been recommended as a semi-permanent restoration for the treatment of large carious lesions and developmental defects such as MIH and amelogenesis imperfecta [10, 11]. SSCs are cost-effective, durable, and require minimal technical sensitivity. However, the major drawback is their unaesthetic appearance, which may be undesirable to some patients [12]. In comparison to deciduous teeth, clinical studies on SSCs in permanent teeth are limited [11, 13, 14]. In one retrospective clinical study [15], the overall success rate for SSCs was reported to be 88% with an almost four-year follow-up. All failed SSCs, on the other hand, were linked to periodontal issues. Permanent SSCs for molars, like primary SSCs, can compromise periodontal health if the crown is over-contoured, has a poor marginal fit, or if cement residue remains in contact with the gingival sulcus, all of which are associated with plaque accumulation [16, 17]. Besides, as reported in a previous study, SSCs do not achieve their maximum adaptation despite contouring and crimping performed to improve their marginal adaptation before cementation [18]. On the other hand, the preformed nature of these kinds of crowns with prefabricated shapes and dimensions makes it difficult to achieve optimal marginal adaptation. Some tricks like reducing the buccal bulge during conventional SSC preparation and using resin-modified glass ionomer cements (GICs) instead of conventional GICs are recommended to reduce marginal discrepancies and hence to prevent microleakage [19]. The most common surface with poor marginal adaptation of SSCs was reported on the buccal surface due to the mesio-buccal bulge creating an under-cut [19]. Although more aggressive preparations are needed for zirconia crowns, they exhibited the lowest internal gap compared to SSCs and pre-veneered SSCs when they cemented with resin-modified GICs, which was attributed to the removal of all coronal bulges during the preparation of ZCs [18].

While efforts are being made to improve crown adaptation and consequently, the longevity of crowns, attempts are also being made to meet aesthetic expectations. Zirconia crowns demonstrated the highest level of aesthetic satisfaction for both parents and patients compared to stainless steel crowns when they were used for primary posterior teeth [20]. More recently, prefabricated zirconia crowns (ZCs) for permanent molars have been introduced as an aesthetic alternative to SSCs in permanent molars. In the adult population, zirconia crowns have a nearly two-decade record of clinical effectiveness. Zirconia is highly biocompatible and has a polished and smooth surface that leads to reduced plaque accumulation and thus less gingival irritation compared to SSCs in primary molars [21]. In a recent invitro study [22], preformed ZCs for permanent molars were found as durable restoration in terms of fracture resistance similar to custom-made Cercon ht Zirconia crowns for adults. Although preformed ZCs for permanent molars were reported as promising in cases involving multiple surface caries, pulp treatment, and malformed teeth such as those affected by MIH [23], currently there is no published research on the clinical effectiveness of ZCs on permanent teeth.

The aim of the present study was to investigate the clinical success of compromised permanent molars in children restored with prefabricated metal crowns or zirconia crowns, and the effects of these crowns on periodontal health. The null hypothesis tested was that there is no difference in periodontal health and clinical success between SSCs and ZCs in young permanent first molars.

## Materials and methods

This was a prospective randomized clinical trial. Both the informed consent form and the study protocol were approved by the Local Ethics Committee (Reg. no: KA-19,056), and registered in the Clinical Trials database (no: NCT05049694, www.clinicaltrials.gov). The study was designed, analyzed, and interpreted in accordance with the Consolidated Standards of Reporting Trials (CONSORT) 2010 checklist. Written informed consent to participate in the study was obtained from all patients and their guardians after they had been informed about and discussed the possible consequences of the treatment.

### Selection of participants

The participants were recruited from patients admitted to a Pediatric Dentistry Department for routine dental treatment between August 2019, and January 2021.

The inclusion criteria were as follows:

1. 6–13-year-old healthy children with at least two fully-erupted permanent first molars showing extensive tissue loss due to multiple (at least three) carious surfaces with or without MIH, which cannot be effectively restored with a direct restoration, e.g. resin composite [12].

## Patients who are willing to participate in the study and attend follow-up appointments

The criteria for exclusion were:

1. Patient with lack of compliance (Grade 1 and 2 patients according to Frankl behavior rating scale).
2. Patients with partially-erupted permanent first molars, or fully-erupted ones without an antagonist.
3. Patients whose teeth have root canal treatment or deep dentin caries with the risk of iatrogenic pulp exposure.
4. Patient with nickel allergy, bruxism and/or deep bite.

**Fig. 1:**
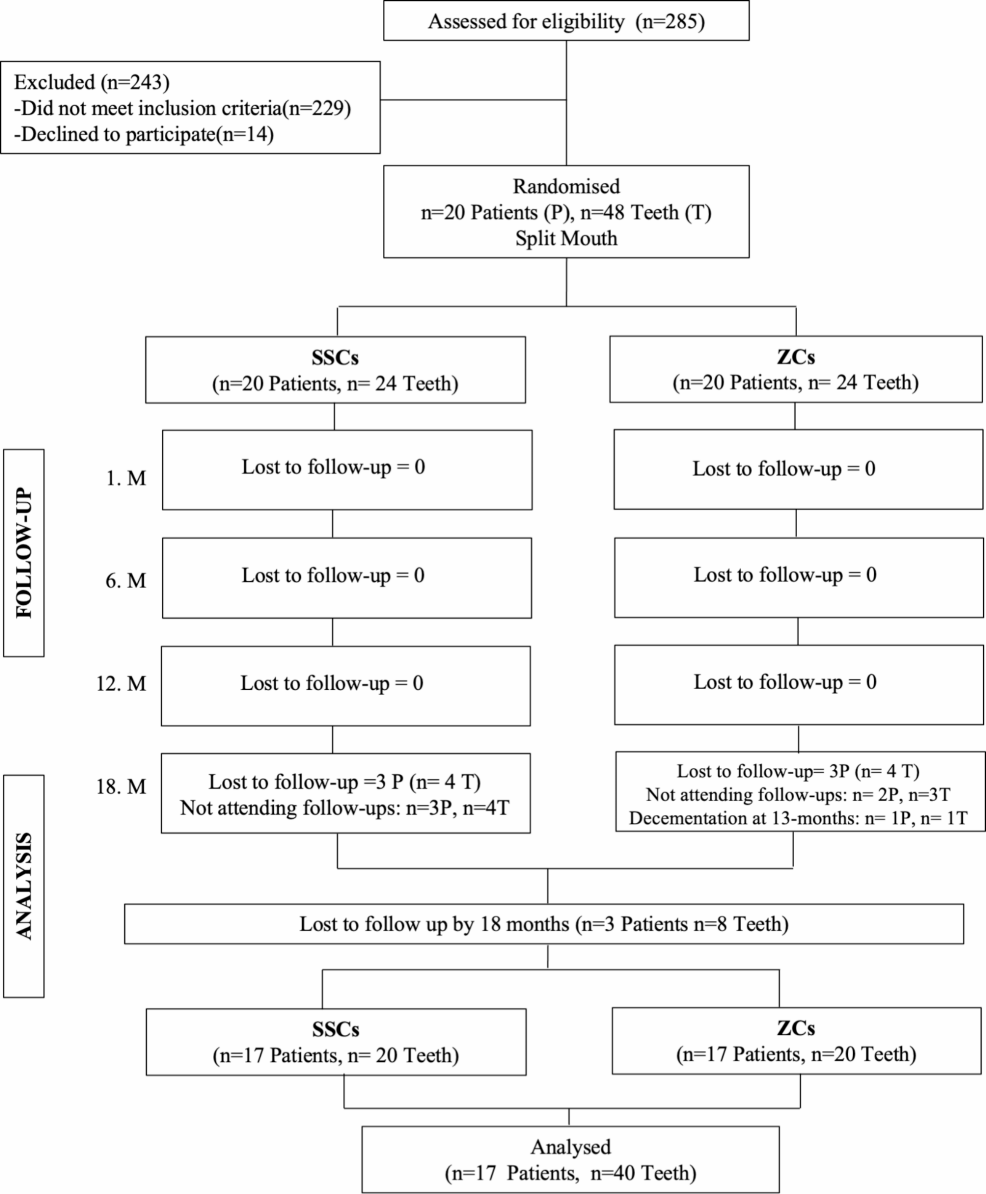
The recruitment and flow diagram of patients

The recruitment and flow diagram of patients is presented in Fig. 1.

### Study design

This was a prospective randomized, controlled trial, using a split-mouth design. Blinding of both the operator and patients was not possible due to the appearance and application procedures of crowns. All treatments were performed by an experienced, calibrated pediatric dentist. Randomization was obtained using a contingency number table on www.random.org and the allocation was kept in sequentially numbered, sealed envelopes.

### Clinical procedures

Forty-eight first permanent molars with extensive tissue loss were included in the study. At least two affected first permanent molars from the same child were randomly assigned to one of the following groups (n = 24/group): group 1; SSC (3 M ESPE, St. Paul, MN), and group 2; ZC (NuSmile Zirconia Pediatric Crowns, Houston, TX).

Local anesthesia was applied and a suitable crown size was determined prior to preparation by measuring the mesiodistal width of the tooth by a caliper. Then the teeth were cleaned with a slow-speed rotary bristle brush.

In the SSC group, the following preparation protocol was used [15]: occlusal reduction, caries removal, interproximal reduction with 5–10-degree taper, rounding of sharp angles and try-in of the SSC. Whenever necessary, the crown margins were trimmed with scissors and/or crimped with crown contouring pliers to achieve a proper gingival adaptation with a snap fit. The teeth were then isolated with cotton rolls, and cemented with glass ionomer (Meron; Voco, Cuxhaven, Germany). The excess cement was quickly wiped off with gauze, and later, all set remnants were removed with a dental explorer and dental floss.

For ZC, the standard preparation protocol was: occlusal reduction and supragingival circumferential reduction, followed by the preparation of a knife-edge subgingival (1-1.5 mm) finishing line with a tapered bur. A pink try-in crown (NuSmile) was used to check the size, passive fit, and occlusion. Then a white crown of the same size was filled with light-cured resin-modified glass ionomer cement (BioCem; NuSmile), and the crown was cemented in place under cotton roll isolation. The crown was tack-cured from buccal and lingual aspects for 5 s to facilitate easy removal of excess cement from the zirconia surface. Then, the final photopolymerization of 40 s was employed from both buccal and lingual aspects. A new LED light-curing unit (Elipar Deep Cure, 3MESPE, MN) was used.

### Clinical assessments

Evaluation of oral hygiene, dental plaque accumulation and gingival health were performed with a periodontal probe and a dental mirror at baseline, 1, 6, 12 and 18 months. Greene and Vermillion simplified oral hygiene index (OHI-S) [24] was used to evaluate oral hygiene. The oral hygiene scores were classified as good (0.0–1.2), fair (1.3–3.0), or poor (> 3.0) [25]. Dental plaque accumulation was assessed with Silness&Löe plaque index (PI) [26], categorized as follows; (0) absence of microbial plaque; (1) thin film of microbial plaque along the free gingival margin; (2) moderate accumulation with plaque in the sulcus; (3) large amount of plaque in sulcus or pocket along the free gingiva margin. The gingival pocket was gently probed with a periodontal probe to assess the gingival health of restored teeth. The situation of gingiva was coded according to Löe&Silness gingival index (GI) [27], with the scores of: (0)-“normal gingiva”; (1)-“mild inflammation: slight change in color and little change in texture”; (2)-“moderate inflammation: moderate glazing, redness, oedema, and hypertrophy; bleeding on pressure”; (3)-“severe inflammation: marked redness and hypertrophy, tendency to spontaneous bleeding”.

For each crown, the following parameters were also evaluated at the recall periods: retention, marginal extension (level) of the crown (supragingival, subgingival and at the gingival margin), marginal adaptation (A: No discrepancies detected with an explorer, B: Detectable discrepancies but clinically acceptable (slight discontinuity in the margin that explorer catches but does not penetrate into any crevice and also radiograph shows a crown ledging < 1 mm), C: Detectable discrepancies (explorer penetrates into buccal, palatal or lingual crevice and also radiograph shows a crown ledging ≥ 1 mm, replacement required) [28], periapical health (defined as radiographic failure in the presence of a radiolucency involving periapical and/or furcation, or internal/external root resorption and widening of the periodontal ligament space) [29], and wear of the antagonist tooth (0: absence of wear, 1: wear only at cusp point, 2: wear at least at the cusps) [30]. The wear of the antagonist tooth was evaluated clinically by visual examination. Intra-oral photos of the crowns were taken at baseline and all follow-up periods. The radiological assessments were performed at 6 and 12 months. For ZCs, discoloration and fracturing/chipping of the crown were also evaluated.

### Statistical analysis

The sample size for the study was calculated using G Power V3.1.8 software based on the results of a previous study [31], with 12nd-month gingival index (GI) scores of 1.56 ± 0.1 (standard deviation) for ZCs and 2.11 ± 0.3 for SSCs. A sample size of 16 (teeth) achieved a power of 80% to detect a difference in gingival indices between the two groups, assuming an effect size of 1.56, using a 2-tailed paired t-test, with a significance level of 0.05. Considering possible dropouts during follow-up, 48 teeth were included in the study, with each group containing at least 24 teeth.

Data were analyzed with SPSS 23.0 software (SPSS Inc., Chicago, IL, USA). Shapiro-Wilk test was used to determine the normality of distribution. Differences between numerical measurements and time points were analysed with one-way analysis of variance for repeated measurements when the assumption of normality was satisfied, otherwise, analysed with the Friedman test. Differences in numerical measurements between crown types were analysed with Student’s t test when the assumption of normality was provided, otherwise, the Mann-Whitney U test was used. Chi-square test, Fisher’s Exact test, or Fisher Freeman-Halton test were used to determine the differences between the categorical variables (e.g., retention, marginal adaptation) of the test groups. Kaplan–Meier analysis was used to evaluate the cumulative survival rates of the crowns.

All clinical assessments were performed by two calibrated pediatric residents. Cohen’s kappa test was used to assess intra- and inter-examiner reliability. In case of disagreement, a consensus scoring was made. For all statistical tests, p < 0.05 was considered statistically significant.

## Results

A total of 20 patients with a mean age of 8 ± 2.49 years were included in the SSC group (n = 24) and ZC group (n = 24). Two patients (6 teeth) were lost to follow-up and one case was lost due to decementation of the ZC at 13th month. Forty permanent first molars of 17 patients (53% girls and 47% boys) were available for evaluation throughout the 18-month follow-up. The intra-examiner reliability for determining gingival health was 0.88 and 0.87, respectively, and the inter-examiner reliability was 0.87.

### Clinical parameters

Over the 18-month follow-up period, none of the crowns showed periapical pathology, wear on antagonist teeth or discoloration. Therefore, these parameters were not included in the statistical analyses. The results of marginal extension and marginal adaptation measurements are shown in Table 1. The cumulative survival rates at 18 months were 95.2% for ZCs and 100% for SSCs. The mean simplified oral health index (OHI-S) of patients was 1.59 ± 0.40 at baseline. There was no significant difference between the median OHI-S scores of teeth at baseline and follow-up examinations (Table 2, p = 0.193).

**Table 1:** Marginal extension and marginal adaptation levels of the crowns

	ZCs	SSCs
	n (%)	n (%)
**Marginal extension of crown (n = 40)**		
A: Subgingival	16 (80)	10 (50)
B: Gingival level	3 (15)	5 (25)
C: Supragingival	1 (5)	5 (25)
**Clinical marginal adaptation (n = 40)**		
A: No discrepancies detected with explorer	20 (100)	19 (95)
B: Detectable discrepancies but clinically acceptable	0 (0)	1(5)
C: Detectable discrepancies, replacement required	0 (0)	0 (0)
**Radiographic marginal adaptation (n = 48)** ^a^		
A: Continuous with the contour of the crown; nice adaptation	20 (83)	23 (96)
B: Slight overhang or under-contour of the crown (< 1 mm ledging) present	4 (17)	1 (4)
C: Crown ledging ≥ 1 mm noted	0 (0)	0 (0)

^a^ Radiographical assessments were performed for 48 crowns at 6- and 12-months

**Table 2:** Time-dependent changes in Simplified Oral Hygiene Index (OHI-S) scores

Month	Simplified Oral Hygiene Index (OHI-S) Scores Median (interquartile range)
**Baseline**	1.50(1.4–1.8)
**1st month**	1.25(1.1–1.8)
**6th month**	1.35(1.1–1.5)
**12th month**	1.30(1.1–1.6)
**18th month**	1.40(1.2–1.5)
p*	0.193

*Repeated Simplified Oral Hygiene Index (OHI-S) Scores were compared using Friedman test

**Fig. 2:**
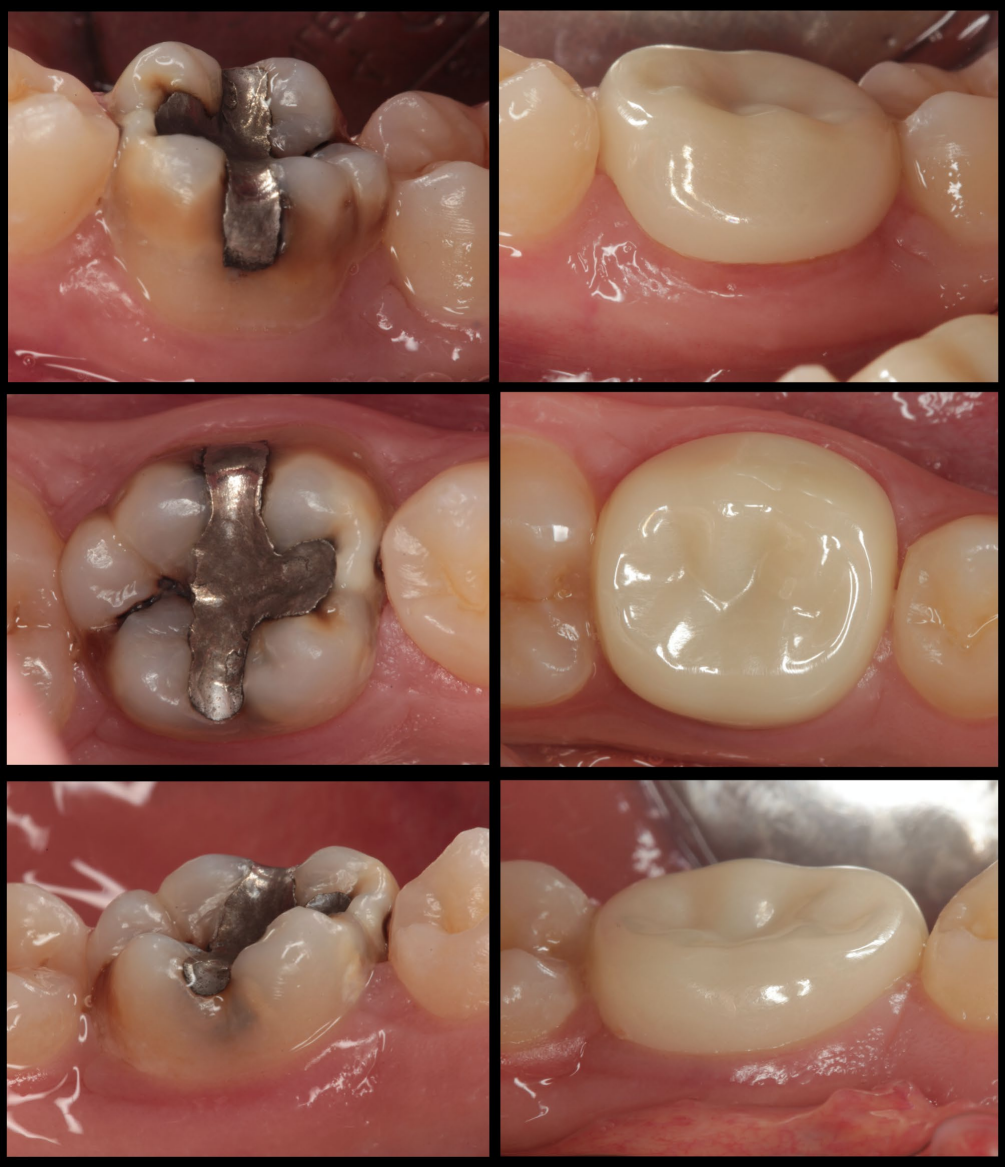
Buccal, occlusal and lingual view of a zirconia crown at 18 months

**Fig. 3:**
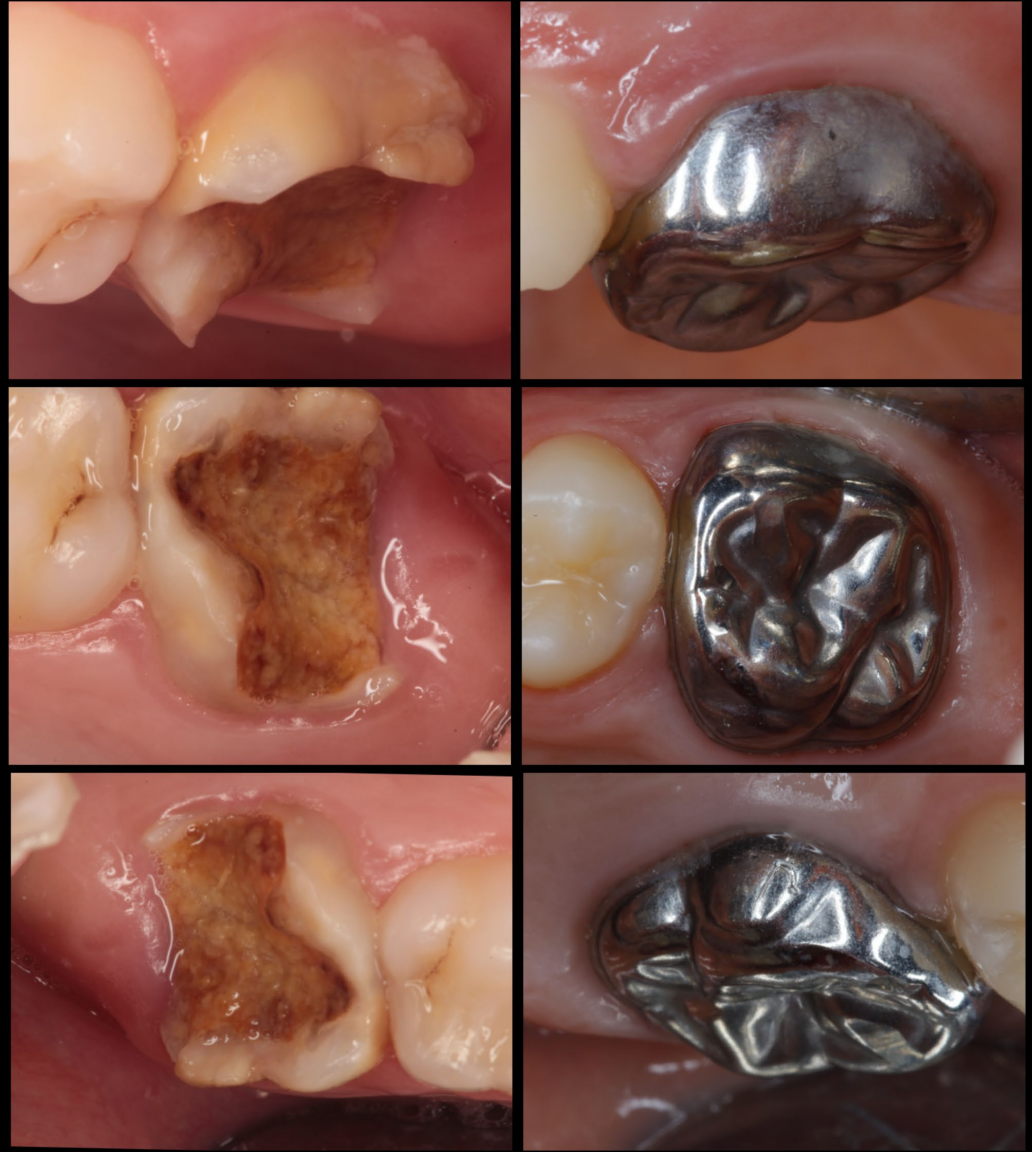
Buccal, occlusal and palatal view of a stainless-steel crown at 18 months

Representative clinical views of permanent ZCs and SSCs before treatment and after 18 months are presented in Figs. 2 and 3, respectively.

### Dental plaque accumulation and gingival health

The PI scores of SSCs remained similar at all follow-up examinations (p = 0.447; Table 3). However, the PI scores of ZCs at follow-ups were significantly lower than those at baseline (p < 0.001; Table 3). Moreover, the PI scores of ZCs were significantly lower than those of SSCs at all recall periods (p < 0.001; Table 3).

**Table 3:** Time-dependent changes in plaque index scores of teeth restored with crowns and intergroup comparisons between SSC and ZC

	Stainless Steel Crown	Zirconia Crown	
**Month**	Plaque index scores Median (Q1, Q3)	Plaque index scores Median (Q1, Q3)	p**
**Baseline**	1.5(1.25–1.75)	1.5(1.25–1.75)	0.362
**1st month**	1.5(1.0-1.75)	0.38(0-0.63)	**< 0.001**
**6th month**	1.5(1.25–1.75)	0.25(0-0.50)	**< 0.001**
**12th month**	1.5(1.25–1.75)	0(0–0)	**< 0.001**
**18th month**	1.625(1.25–1.75)	0(0–0)	**< 0.001**
p*	0.447	**< 0.001**	

* Repeated plaque index scores were compared using Friedman test

** Plaque index scores of crowns were compared using Mann-Whitney U test

GI scores of SSCs were significantly lower at 18 months than those at 1, 6, and 12 months (p = 0.024, p = 0.012 and p = 0.032, respectively). The GI scores at follow-ups were higher than baseline, but the difference was statistically significant only at 6 months (p = 0.032). The GI scores of ZCs were significantly different among the recall periods (p < 0.001, Table 4). Pairwise comparisons showed that GI scores at 18 months were significantly lower than those at baseline, 1-month, and 6-month recalls (p = 0.037, p = 0.024, p < 0.001, respectively). The GI scores of ZCs were significantly lower than those of SSCs at 6-, 12- and 18-month follow-ups (p = 0.006, p < 0.001 and p < 0.001, respectively, Table 4).

**Table 4:** Time-dependent changes in gingival index scores of teeth restored with crowns and intergroup comparisons between SSC and ZC.

	Stainless Steel Crown	Zirconia Crown	
**Month**	Gingival index scores Median (Q1, Q3)	Gingival index scores Median (Q1, Q3)	p***
**Baseline**	1.125(0.88–1.50)	1.25(1.0-1.38)	0.948^a^
**1st month**	1.5(1.0-1.75)	1.0(0.75–1.75)	0.100^b^
**6th month**	1.5(1.25–1.75)	0.125(1.0-1.50)	**0.006** ^**a**^
**12th month**	1.375(1.25–1.75)	0.875(0.50–1.25)	**< 0.001** ^**b**^
**18th month**	1.25(0.88–1.50)	0.5(0.25-1.0)	**< 0.001** ^**a**^
**p**	0.014*	< 0.001**	

* Stainless steel crown repeated gingival index scores were compared using Friedman test

**Zirconia crown repeated gingival index scores were compared using one way analysis of variance for repeated measures due to the assumption of normality was satisfied

*** Gingival index scores of crowns were compared using Mann-Whitney U test: ^a^ p values were obtained using Student t test, ^b^: p values were obtained using Mann-Whitney U test

## Discussion

Full coverage restoration of permanent molars with SSCs has been recommended for the treatment of extensive multi-surface caries, developmental enamel and dentinal defects, carious partially erupted molars and after endodontic treatment [10, 32]. Compared to multi-surface resin composite restorations, SSCs exhibit better longevity and less recurrent caries [10, 32]. In the present study, all SSCs remained in function with no recurrent caries or periapical pathology. Sigal et al. [32] compared the long-term clinical outcomes of SSCs with amalgam restorations in a special-needs population, and reported a 10-year survival rate of 79.2% and 63.5%, for new SSCs and amalgam restorations, respectively. In two retrospective studies conducted on permanent molars, the 5-year survival rate of 115 SSCs was 82.8% [14] and the 45-month survival rate of 155 crowns was 88% [15]. All of these findings support the idea that SSCs are a long-lasting restorative option for compromised permanent first molars.

Despite those advantages, however, the use of SSCs in primary molars appears to be more common than in permanent molars [13, 32], and there have been concerns regarding the use of SSCs in permanent molars [13, 33], mainly due to their possible periodontal impacts. A recent study [13] showed an increase in gingival index scores and counts of periodontal pathogens around SSCs six months after placement on permanent first molars. Likewise, Chen et al. [34] reported an increase in gingival inflammation and plaque accumulation around SSCs that were used to restore permanent molars affected by amelogenesis imperfecta. In their study, 33% (9/27) of SSCs had marginal discrepancy, which may contribute to plaque retention. Placing the SSCs subgingivally may also pose a risk for violation of the biological width of the periodontal attachment. On the other hand, it is often necessary to place SSCs subgingivally, especially in molars with large carious lesions extending subgingivally or those that have partially erupted. Finally, both the patient and their parents may have aesthetic concerns due to the metallic appearance of SSCs, even in the posterior area [33]. ZCs for primary teeth have been developed as an aesthetic alternative to SSCs. Primary ZCs have shown successful clinical outcomes in terms of esthetics, clinical retention and gingival health [21, 35]. ZCs do not require marginal adjustment, so their smooth, glazed, and polished surface remains protected and lowers the surface roughness and energy [31], whereas trimming, cutting, and crimping of the SSCs is a significant risk factor for plaque accumulation on SSCs [31]. Mathew et al.’s 12-month investigation of primary molars repaired with ZCs and SSCs [31], revealed lower adherence of Streptococcus mutans on ZC surfaces than on SSC surfaces, as well as significantly reduced gingival irritation and plaque buildup around ZCs.

More recently, preformed zirconia crowns for permanent molars have been introduced as an esthetic alternative to permanent molar SSCs. Although many studies are present comparing the SSCs and ZCs on primary molars, to the best of our knowledge, no clinical trial has been published on the clinical performance of permanent molar ZCs. Permanent molar ZCs can be utilized in partially erupted molars and can be installed in a single session without the requirement for analog or digital impressions, in addition to the inherent benefits of primary molar ZCs [23]. In the present study, the cumulative survival rates at 18 months were 95.2% for permanent molar ZCs and 100% for SSCs. Only one preformed ZC became decemented at 12 months, leading to a decrease in the 18-month survival rate of ZC compared to the SSCs. Zirconia crowns require a passive fit and hence necessitate significantly more tooth reduction than SSCs [36]. The type of luting cement, adequate preparation, occlusal convergence angles (taper), and remaining clinical crown height are additional variables that affect retention [37]. Crimping can increase the retention of SSCs at the expense of decreasing marginal accuracy, but it cannot be applied to ZCs due to their lack of malleability and elasticity [38]. Jing et al. [37] reported that the occluso-cervical height of the remaining crown preparation should be at least two millimeters for the retention of EZ Crowns (Sprig, CA, U.S.A.), which is a different zirconia crown brand. Although the remaining crown height was not evaluated in the present study, a high retention rate was observed for permanent molar ZCs at 18 months. Here, both the GI and PI scores of ZCs were significantly lower than those of SSCs. ZCs also exhibited better plaque and gingival index scores compared to pretreatment values, while SSCs showed higher plaque and gingival index values. These results are consistent with several clinical trials conducted in primary teeth [21, 31]. According to Sharaf et al. [17], SSCs have no direct effect on gingival health or interproximal bone levels, indicating that the oral hygiene level is the main risk factor. The patients’ oral hygiene could not be improved throughout the current investigation, despite frequent recalls and patient encouragement. In the presence of fair oral hygiene, ZCs showed better periodontal health than SSCs in the same patients.

In the present study, the crowns were placed and assessed during the COVID-19 pandemic. Even though this circumstance had an impact on the study’s intended sample size, the achieved sample size was still adequate based on the estimation with 80% power. Restorative treatments have dramatically decreased during the pandemic [39] due to the aerosol they produce, leading to an increased number of patients with compromised permanent molars. The favorable short-term results of the present study may contribute to the knowledge of the management of compromised permanent molars with preformed crowns.

The results of the present study should be assessed along with a number of limitations. First, the examiners could not be blinded due to the color difference of the crowns. Second, Covid-19 pandemic caused challenges in recruiting additional patients who met the inclusion criteria and accepted participating in the study, likewise other clinical trials. Another limitation was that the indications for SSCs and ZCs might be different since tooth preparation is more extensive for zirconium crowns which required more patient compliance. Neither crown type led to visible, clinically-detectable wear on antagonist tooth, but measuring the quantification of enamel wear with three dimensional (3D) techniques [40] is required for more reliable outcomes. Although, this study included only cooperative patients, the remaining crown height should also be evaluated to elucidate its possible effect on clinical retention. In this study, a lower sample size was obtained from the registered protocol in Clinical Trials (NCT05049694), but the sample size of 48 was found sufficient for the present study’s validation. Further clinical trials with greater sample sizes and longer follow-up periods are required to provide evidence for long-term survival. Finally, it would be interesting to compare SSCs and ZCs in children with better oral hygiene to eliminate the possible effect of poor oral hygiene. Nevertheless, the present results are encouraging, and provide a favorable short-term outcome of SSCs and ZCs applications in compromised permanent molars.

## Conclusions

This study has shown that preformed permanent molar ZCs exhibit significantly lower plaque accumulation and gingival inflammation than permanent molars SSCs in young permanent first molars. Both types of crowns can be used in compromised permanent molars with successful clinical outcomes.

## Data Availability

The datasets used and/or analyzed during the current study available from the corresponding author on reasonable request.

## Abbreviations

FPMs: First permanent molars
MIH: Molar-incisor hypomineralisation
SSCs: Stainless-steel crowns
ZCs: Zirconia crowns
CONSORT: Consolidated Standards of Reporting Trials
GI: Gingival index
PI: Plaque index
OHI-S: Simplified oral hygiene index
